# P05 The inaugural Infection Summit at Leeds Teaching Hospitals NHS Trust (LTHT)

**DOI:** 10.1093/jacamr/dlae217.009

**Published:** 2025-01-23

**Authors:** Zara Tariq, Jessica Martin, Emma O’Cofaigh, Kelly Atack, Kavita Sethi, Wayne Short, Stephen Bush, Chris Day, Fiona Byrne, Sarah Hackney, Lam Nguyen, Shazia Nazir

**Affiliations:** Leeds Teaching Hospitals NHS Trust, Leeds, UK; Leeds Teaching Hospitals NHS Trust, Leeds, UK; Leeds Teaching Hospitals NHS Trust, Leeds, UK; Leeds Teaching Hospitals NHS Trust, Leeds, UK; Leeds Teaching Hospitals NHS Trust, Leeds, UK; Leeds Teaching Hospitals NHS Trust, Leeds, UK; Leeds Teaching Hospitals NHS Trust, Leeds, UK; Leeds Teaching Hospitals NHS Trust, Leeds, UK; Leeds Teaching Hospitals NHS Trust, Leeds, UK; Leeds Teaching Hospitals NHS Trust, Leeds, UK; Leeds Teaching Hospitals NHS Trust, Leeds, UK; Leeds Teaching Hospitals NHS Trust, Leeds, UK

## Abstract

**Objectives:**

To establish our first Infection Summit, aiming to engage and educate non-infection specialists to locally improve antimicrobial stewardship (AMS) and to identify quality improvement (QI) ideas and AMS interventions, through collaboration. To also gather feedback and determine any benefits of this trust-wide event.

**Methods:**

LTHT did not meet the NHS standard contract targets for antimicrobial consumption in 2023–24 and so the Infection Summit was created and implemented. A wide variety of healthcare professionals from 14 different clinical service units (CSUs) attended. This was essential as LTHT is one of the largest teaching hospitals in Europe and AMS can vary trust wide. The event was held in-person and online, with 89 attendees, to allow equitable accessibility. The day consisted of educational presentations delivered by AMS experts, on a range of topics: point prevalence survey data, infection prevention, blood culture pathway, IV access devices, guideline and documentation digital innovations and an AMS global institutional partnership. The afternoon QI workshops focused on devices, diagnostics and digital AMS. Post summit, feedback was gathered via an electronic, anonymous and optional survey, consisting of quantitative and qualitative questions. It concentrated on the content and structure of the day, and how attendees felt about their knowledge and practice in AMS. We did acquire some feedback around barriers to changing behaviour relating to AMS s and how attendees could overcome them.

**Results:**

The survey had a 51% (*n*=45) overall response rate, including various healthcare professionals: nurses, medics, pharmacy and other healthcare professionals. The average, overall experience rating for this event was 4.5 stars out of 5. Ninety-six percent of attendees felt their knowledge and understanding of AMS increased, 89% felt their attendance would improve patient outcomes. Eighty-nine percent also felt empowered to implement local QI ideas and 84% were encouraged to collaborate with other CSUs. Fifty-eight percent of attendees would change the management and/or treatment of their patients, 38% wanted to change certain protocols, policies and/or procedures and 11% felt the summit validated their current practice. Only 2 attendees (4%) felt no change in their practice was needed. Based on their experience of attending the first summit, 73% of attendees stated they would be extremely like to attend, 11% would likely attend and 16% were unlikely to attend. The qualitative themes included improvement suggestions for future summits and how they would overcome existing barriers to implementing better AMS in their areas. Colleagues felt the collaboration, discussion and education were the most effective aspects of this event.

**Conclusions:**

This feedback highlights the success of this trust-wide educational AMS event and the need for future events. It confirms that attendees who completed the survey felt it was a beneficial event for their patients and will lead to improved outcomes. The QI workshops generated over 35 ideas, and we will hold a follow-up event, to support the development of these workstreams and champion any implemented interventions. Additionally, this will further promote interprofessional working and collaborative learning and improvement to share best practice in improving AMS at LTHT.

